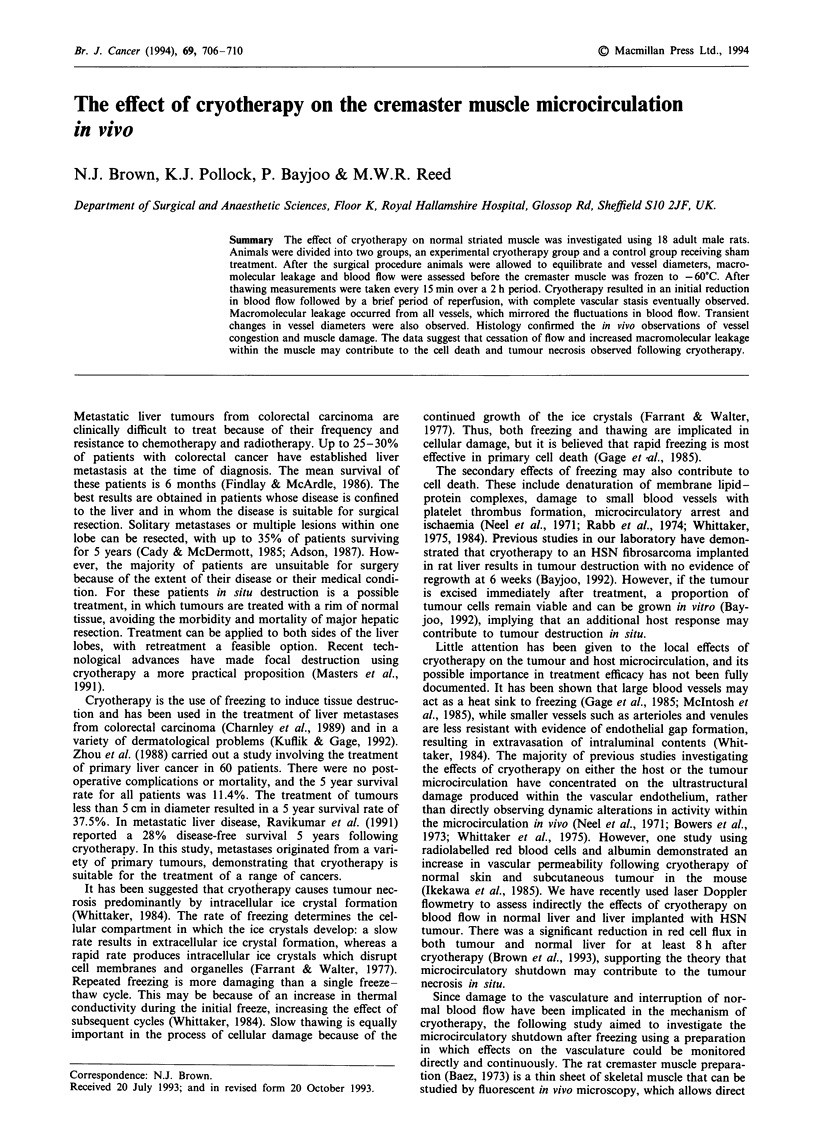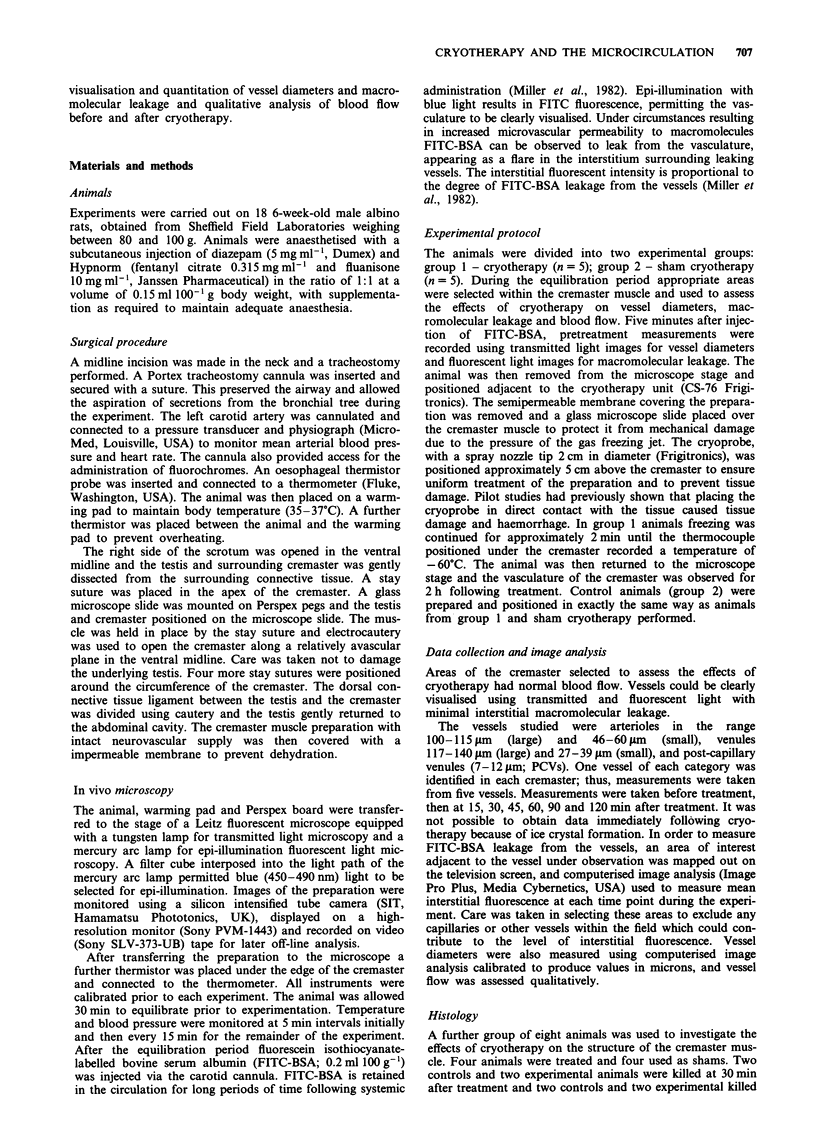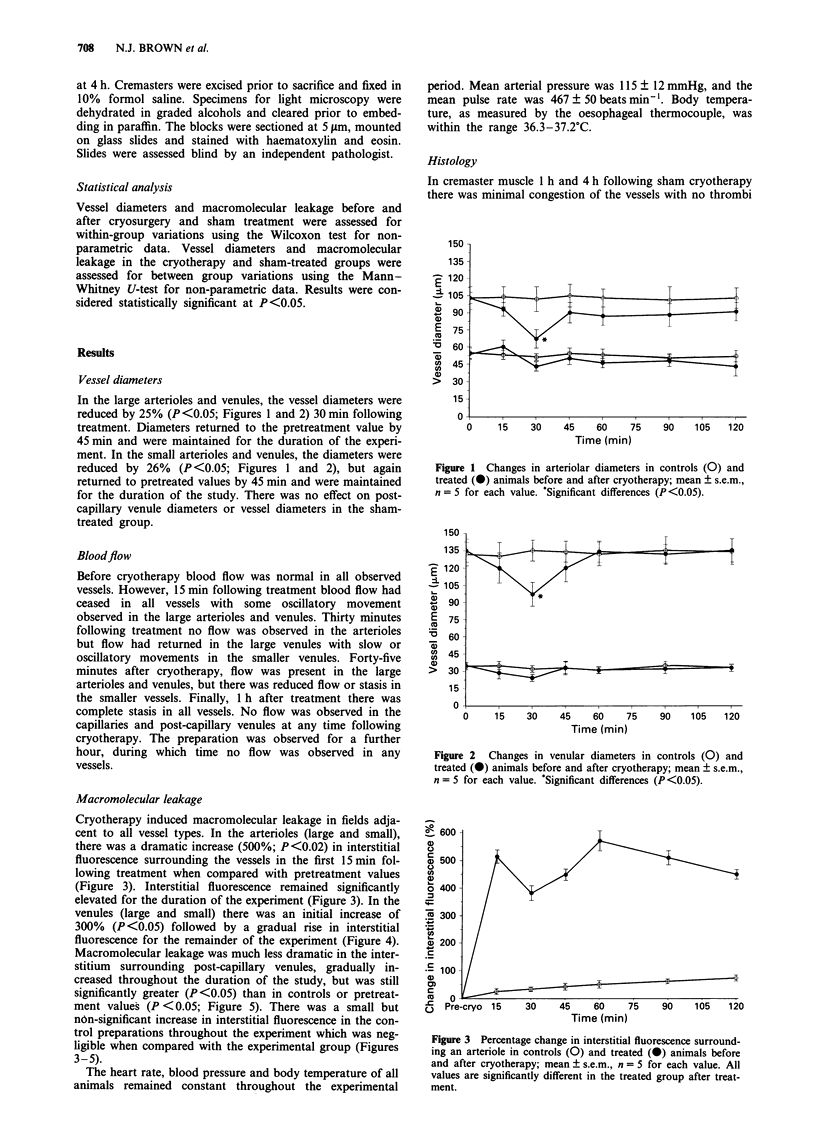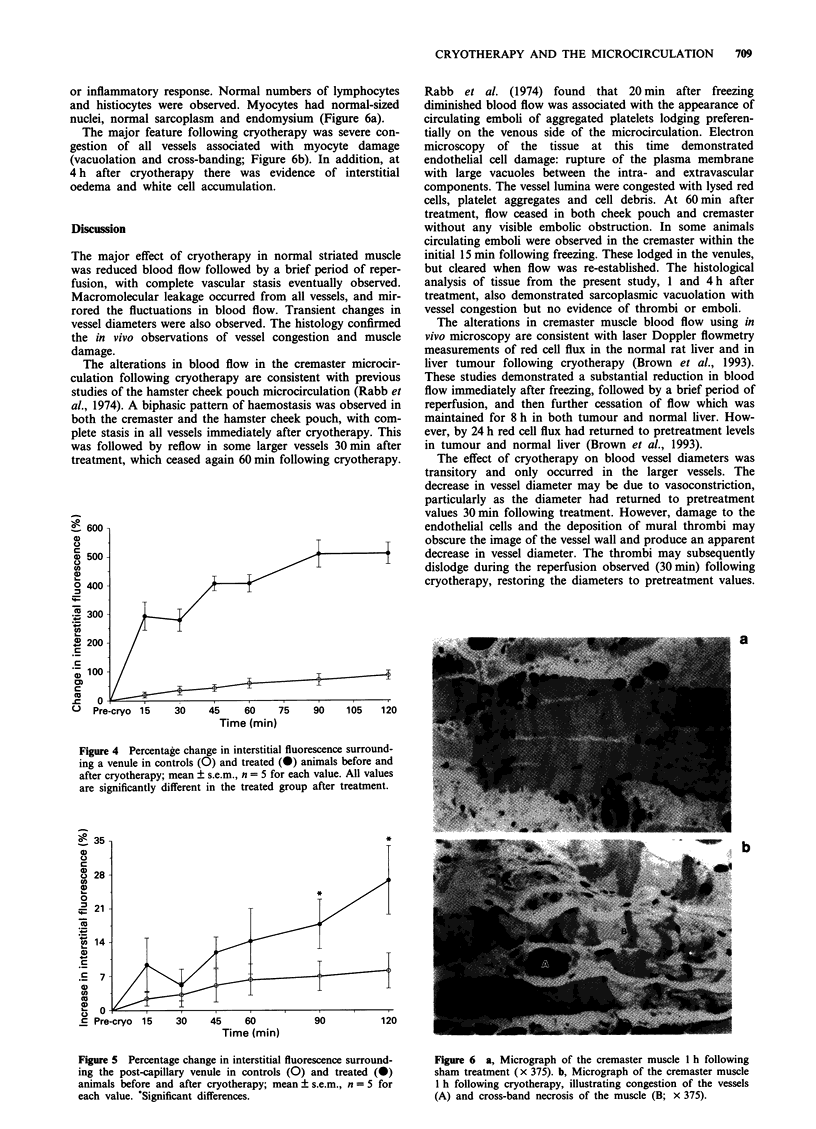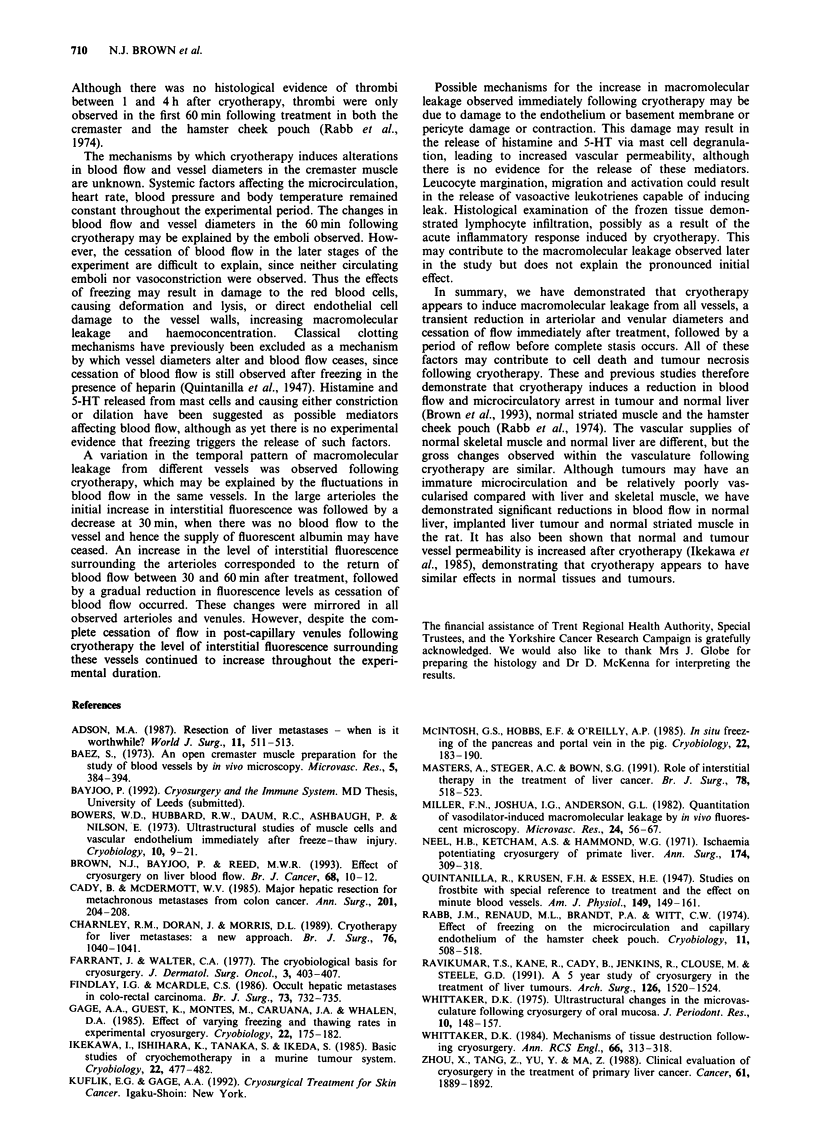# The effect of cryotherapy on the cremaster muscle microcirculation in vivo.

**DOI:** 10.1038/bjc.1994.133

**Published:** 1994-04

**Authors:** N. J. Brown, K. J. Pollock, P. Bayjoo, M. W. Reed

**Affiliations:** Department of Surgical and Anaesthetic Sciences, Royal Hallamshire Hospital, Sheffield, UK.

## Abstract

**Images:**


Br. J. Cancer (1994), 69, 706–710 © Macmillan Press Ltd., 1994

### The effect of cryotherapy on the cremaster muscle microcirculation *in vivo*

N.J. Brown, K.J. Pollock, P. Bayjoo & M.W.R. Reed

*Department of Surgical and Anaesthetic Sciences, Floor K, Royal Hallamshire Hospital, Glossop Rd, Sheffield S10 2JF, UK.*

**Summary** The effect of cryotherapy on normal striated muscle was investigated using 18 adult male rats. Animals were divided into two groups, an experimental cryotherapy group and a control group receiving sham treatment. After the surgical procedure animals were allowed to equilibrate and vessel diameters, macromolecular leakage and blood flow were assessed before the cremaster muscle was frozen to −60°C. After thawing measurements were taken every 15 min over a 2 h period. Cryotherapy resulted in an initial reduction in blood flow followed by a brief period of reperfusion, with complete vascular stasis eventually observed. Macromolecular leakage occurred from all vessels, which mirrored the fluctuations in blood flow. Transient changes in vessel diameters were also observed. Histology confirmed the *in vivo* observations of vessel congestion and muscle damage. The data suggest that cessation of flow and increased macromolecular leakage within the muscle may contribute to the cell death and tumour necrosis observed following cryotherapy.

Metastatic liver tumours from colorectal carcinoma are clinically difficult to treat because of their frequency and resistance to chemotherapy and radiotherapy. Up to 25–30% of patients with colorectal cancer have established liver metastasis at the time of diagnosis. The mean survival of these patients is 6 months (Findlay & McArdle, 1986). The best results are obtained in patients whose disease is confined to the liver and in whom the disease is suitable for surgical resection. Solitary metastases or multiple lesions within one lobe can be resected, with up to 35% of patients surviving for 5 years (Cady & McDermott, 1985; Adson, 1987). However, the majority of patients are unsuitable for surgery because of the extent of their disease or their medical condition. For these patients *in situ* destruction is a possible treatment, in which tumours are treated with a rim of normal tissue, avoiding the morbidity and mortality of major hepatic resection. Treatment can be applied to both sides of the liver lobes, with retreatment a feasible option. Recent technological advances have made focal destruction using cryotherapy a more practical proposition (Masters *et al.*, 1991).

Cryotherapy is the use of freezing to induce tissue destruction and has been used in the treatment of liver metastases from colorectal carcinoma (Charnley *et al.*, 1989) and in a variety of dermatological problems (Kuflik & Gage, 1992). Zhou *et al.* (1988) carried out a study involving the treatment of primary liver cancer in 60 patients. There were no postoperative complications or mortality, and the 5 year survival rate for all patients was 11.4%. The treatment of tumours less than 5 cm in diameter resulted in a 5 year survival rate of 37.5%. In metastatic liver disease, Ravikumar *et al.* (1991) reported a 28% disease-free survival 5 years following cryotherapy. In this study, metastases originated from a variety of primary tumours, demonstrating that cryotherapy is suitable for the treatment of a range of cancers.

It has been suggested that cryotherapy causes tumour necrosis predominantly by intracellular ice crystal formation (Whittaker, 1984). The rate of freezing determines the cellular compartment in which the ice crystals develop: a slow rate results in extracellular ice crystal formation, whereas a rapid rate produces intracellular ice crystals which disrupt cell membranes and organelles (Farrant & Walter, 1977). Repeated freezing is more damaging than a single freeze–thaw cycle. This may be because of an increase in thermal conductivity during the initial freeze, increasing the effect of subsequent cycles (Whittaker, 1984). Slow thawing is equally important in the process of cellular damage because of the continued growth of the ice crystals (Farrant & Walter, 1977). Thus, both freezing and thawing are implicated in cellular damage, but it is believed that rapid freezing is most effective in primary cell death (Gage *et al.*, 1985).

The secondary effects of freezing may also contribute to cell death. These include denaturation of membrane lipid–protein complexes, damage to small blood vessels with platelet thrombus formation, microcirculatory arrest and ischaemia (Neel *et al.*, 1971; Rabb *et al.*, 1974; Whittaker, 1975, 1984). Previous studies in our laboratory have demonstrated that cryotherapy to an HSN fibrosarcoma implanted in rat liver results in tumour destruction with no evidence of regrowth at 6 weeks (Bayjoo, 1992). However, if the tumour is excised immediately after treatment, a proportion of tumour cells remain viable and can be grown *in vitro* (Bayjoo, 1992), implying that an additional host response may contribute to tumour destruction *in situ*.

Little attention has been given to the local effects of cryotherapy on the tumour and host microcirculation, and its possible importance in treatment efficacy has not been fully documented. It has been shown that large blood vessels may act as a heat sink to freezing (Gage *et al.*, 1985; McIntosh *et al.*, 1985), while smaller vessels such as arterioles and venules are less resistant with evidence of endothelial gap formation, resulting in extravasation of intraluminal contents (Whittaker, 1984). The majority of previous studies investigating the effects of cryotherapy on either the host or the tumour microcirculation have concentrated on the ultrastructural damage produced within the vascular endothelium, rather than directly observing dynamic alterations in activity within the microcirculation *in vivo* (Neel *et al.*, 1971; Bowers *et al.*, 1973; Whittaker *et al.*, 1975). However, one study using radiolabelled red blood cells and albumin demonstrated an increase in vascular permeability following cryotherapy of normal skin and subcutaneous tumour in the mouse (Ikekawa *et al.*, 1985). We have recently used laser Doppler flowmetry to assess indirectly the effects of cryotherapy on blood flow in normal liver and liver implanted with HSN tumour. There was a significant reduction in red cell flux in both tumour and normal liver for at least 8 h after cryotherapy (Brown *et al.*, 1993), supporting the theory that microcirculatory shutdown may contribute to the tumour necrosis *in situ*.

Since damage to the vasculature and interruption of normal blood flow have been implicated in the mechanism of cryotherapy, the following study aimed to investigate the microcirculatory shutdown after freezing using a preparation in which effects on the vasculature could be monitored directly and continuously. The rat cremaster muscle preparation (Baez, 1973) is a thin sheet of skeletal muscle that can be studied by fluorescent *in vivo* microscopy, which allows direct

Correspondence: N.J. Brown.
Received 20 July 1993; and in revised form 20 October 1993.

visualisation and quantitation of vessel diameters and macromolecular leakage and qualitative analysis of blood flow before and after cryotherapy.

### Materials and methods

### *Animals*

Experiments were carried out on 18 6-week-old male albino rats, obtained from Sheffield Field Laboratories weighing between 80 and 100 g. Animals were anaesthetised with a subcutaneous injection of diazepam (5 mg $ml^{-1}$, Dumex) and Hypnorm (fentanyl citrate 0.315 mg $ml^{-1}$ and fluanisone 10 mg $ml^{-1}$, Janssen Pharmaceutical) in the ratio of 1:1 at a volume of 0.15 ml $100^{-1}$ g body weight, with supplementation as required to maintain adequate anaesthesia.

### *Surgical procedure*

A midline incision was made in the neck and a tracheostomy performed. A Portex tracheostomy cannula was inserted and secured with a suture. This preserved the airway and allowed the aspiration of secretions from the bronchial tree during the experiment. The left carotid artery was cannulated and connected to a pressure transducer and physiograph (Micro-Med, Louisville, USA) to monitor mean arterial blood pressure and heart rate. The cannula also provided access for the administration of fluorochromes. An oesophageal thermistor probe was inserted and connected to a thermometer (Fluke, Washington, USA). The animal was then placed on a warming pad to maintain body temperature (35–37°C). A further thermistor was placed between the animal and the warming pad to prevent overheating.

The right side of the scrotum was opened in the ventral midline and the testis and surrounding cremaster was gently dissected from the surrounding connective tissue. A stay suture was placed in the apex of the cremaster. A glass microscope slide was mounted on Perspex pegs and the testis and cremaster positioned on the microscope slide. The muscle was held in place by the stay suture and electrocautery was used to open the cremaster along a relatively avascular plane in the ventral midline. Care was taken not to damage the underlying testis. Four more stay sutures were positioned around the circumference of the cremaster. The dorsal connective tissue ligament between the testis and the cremaster was divided using cautery and the testis gently returned to the abdominal cavity. The cremaster muscle preparation with intact neurovascular supply was then covered with a impermeable membrane to prevent dehydration.

### In vivo *microscopy*

The animal, warming pad and Perspex board were transferred to the stage of a Leitz fluorescent microscope equipped with a tungsten lamp for transmitted light microscopy and a mercury arc lamp for epi-illumination fluorescent light microscopy. A filter cube interposed into the light path of the mercury arc lamp permitted blue (450–490 nm) light to be selected for epi-illumination. Images of the preparation were monitored using a silicon intensified tube camera (SIT, Hamamatsu Phototonics, UK), displayed on a high-resolution monitor (Sony PVM-1443) and recorded on video (Sony SLV-373-UB) tape for later off-line analysis.

After transferring the preparation to the microscope a further thermistor was placed under the edge of the cremaster and connected to the thermometer. All instruments were calibrated prior to each experiment. The animal was allowed 30 min to equilibrate prior to experimentation. Temperature and blood pressure were monitored at 5 min intervals initially and then every 15 min for the remainder of the experiment. After the equilibration period fluorescein isothiocyanate-labelled bovine serum albumin (FITC-BSA; 0.2 ml 100 $g^{-1}$) was injected via the carotid cannula. FITC-BSA is retained in the circulation for long periods of time following systemic administration (Miller *et al.*, 1982). Epi-illumination with blue light results in FITC fluorescence, permitting the vasculature to be clearly visualised. Under circumstances resulting in increased microvascular permeability to macromolecules FITC-BSA can be observed to leak from the vasculature, appearing as a flare in the interstitium surrounding leaking vessels. The interstitial fluorescent intensity is proportional to the degree of FITC-BSA leakage from the vessels (Miller *et al.*, 1982).

### *Experimental protocol*

The animals were divided into two experimental groups: group 1 – cryotherapy ($n = 5$); group 2 – sham cryotherapy ($n = 5$). During the equilibration period appropriate areas were selected within the cremaster muscle and used to assess the effects of cryotherapy on vessel diameters, macromolecular leakage and blood flow. Five minutes after injection of FITC-BSA, pretreatment measurements were recorded using transmitted light images for vessel diameters and fluorescent light images for macromolecular leakage. The animal was then removed from the microscope stage and positioned adjacent to the cryotherapy unit (CS-76 Frigitronics). The semipermeable membrane covering the preparation was removed and a glass microscope slide placed over the cremaster muscle to protect it from mechanical damage due to the pressure of the gas freezing jet. The cryoprobe, with a spray nozzle tip 2 cm in diameter (Frigitronics), was positioned approximately 5 cm above the cremaster to ensure uniform treatment of the preparation and to prevent tissue damage. Pilot studies had previously shown that placing the cryoprobe in direct contact with the tissue caused tissue damage and haemorrhage. In group 1 animals freezing was continued for approximately 2 min until the thermocouple positioned under the cremaster recorded a temperature of −60°C. The animal was then returned to the microscope stage and the vasculature of the cremaster was observed for 2 h following treatment. Control animals (group 2) were prepared and positioned in exactly the same way as animals from group 1 and sham cryotherapy performed.

### *Data collection and image analysis*

Areas of the cremaster selected to assess the effects of cryotherapy had normal blood flow. Vessels could be clearly visualised using transmitted and fluorescent light with minimal interstitial macromolecular leakage.

The vessels studied were arterioles in the range 100–115 μm (large) and 46–60 μm (small), venules 117–140 μm (large) and 27–39 μm (small), and post-capillary venules (7–12 μm; PCVs). One vessel of each category was identified in each cremaster; thus, measurements were taken from five vessels. Measurements were taken before treatment, then at 15, 30, 45, 60, 90 and 120 min after treatment. It was not possible to obtain data immediately following cryotherapy because of ice crystal formation. In order to measure FITC-BSA leakage from the vessels, an area of interest adjacent to the vessel under observation was mapped out on the television screen, and computerised image analysis (Image Pro Plus, Media Cybernetics, USA) used to measure mean interstitial fluorescence at each time point during the experiment. Care was taken in selecting these areas to exclude any capillaries or other vessels within the field which could contribute to the level of interstitial fluorescence. Vessel diameters were also measured using computerised image analysis calibrated to produce values in microns, and vessel flow was assessed qualitatively.

### *Histology*

A further group of eight animals was used to investigate the effects of cryotherapy on the structure of the cremaster muscle. Four animals were treated and four used as shams. Two controls and two experimental animals were killed at 30 min after treatment and two controls and two experimental killed at 4 h. Cremasters were excised prior to sacrifice and fixed in 10% formol saline. Specimens for light microscopy were dehydrated in graded alcohols and cleared prior to embedding in paraffin. The blocks were sectioned at 5 μm, mounted on glass slides and stained with haematoxylin and eosin. Slides were assessed blind by an independent pathologist.

*Statistical analysis*

Vessel diameters and macromolecular leakage before and after cryosurgery and sham treatment were assessed for within-group variations using the Wilcoxon test for non-parametric data. Vessel diameters and macromolecular leakage in the cryotherapy and sham-treated groups were assessed for between group variations using the Mann–Whitney *U*-test for non-parametric data. Results were considered statistically significant at $P<0.05$.

### Results

*Vessel diameters*

In the large arterioles and venules, the vessel diameters were reduced by 25% ($P<0.05$; Figures 1 and 2) 30 min following treatment. Diameters returned to the pretreatment value by 45 min and were maintained for the duration of the experiment. In the small arterioles and venules, the diameters were reduced by 26% ($P<0.05$; Figures 1 and 2), but again returned to pretreated values by 45 min and were maintained for the duration of the study. There was no effect on post-capillary venule diameters or vessel diameters in the sham-treated group.

*Blood flow*

Before cryotherapy blood flow was normal in all observed vessels. However, 15 min following treatment blood flow had ceased in all vessels with some oscillatory movement observed in the large arterioles and venules. Thirty minutes following treatment no flow was observed in the arterioles but flow had returned in the large venules with slow or oscillatory movements in the smaller venules. Forty-five minutes after cryotherapy, flow was present in the large arterioles and venules, but there was reduced flow or stasis in the smaller vessels. Finally, 1 h after treatment there was complete stasis in all vessels. No flow was observed in the capillaries and post-capillary venules at any time following cryotherapy. The preparation was observed for a further hour, during which time no flow was observed in any vessels.

*Macromolecular leakage*

Cryotherapy induced macromolecular leakage in fields adjacent to all vessel types. In the arterioles (large and small), there was a dramatic increase (500%; $P<0.02$) in interstitial fluorescence surrounding the vessels in the first 15 min following treatment when compared with pretreatment values (Figure 3). Interstitial fluorescence remained significantly elevated for the duration of the experiment (Figure 3). In the venules (large and small) there was an initial increase of 300% ($P<0.05$) followed by a gradual rise in interstitial fluorescence for the remainder of the experiment (Figure 4). Macromolecular leakage was much less dramatic in the interstitium surrounding post-capillary venules, gradually increased throughout the duration of the study, but was still significantly greater ($P<0.05$) than in controls or pretreatment values ($P<0.05$; Figure 5). There was a small but non-significant increase in interstitial fluorescence in the control preparations throughout the experiment which was negligible when compared with the experimental group (Figures 3–5).

The heart rate, blood pressure and body temperature of all animals remained constant throughout the experimental period. Mean arterial pressure was 115 ± 12 mmHg, and the mean pulse rate was 467 ± 50 beats $min^{-1}$. Body temperature, as measured by the oesophageal thermocouple, was within the range 36.3–37.2°C.

*Histology*

In cremaster muscle 1 h and 4 h following sham cryotherapy there was minimal congestion of the vessels with no thrombi

**Figure 1** Changes in arteriolar diameters in controls (○) and treated (●) animals before and after cryotherapy; mean ± s.e.m., $n=5$ for each value. *Significant differences ($P<0.05$).

**Figure 2** Changes in venular diameters in controls (○) and treated (●) animals before and after cryotherapy; mean ± s.e.m., $n=5$ for each value. *Significant differences ($P<0.05$).

**Figure 3** Percentage change in interstitial fluorescence surrounding an arteriole in controls (○) and treated (●) animals before and after cryotherapy; mean ± s.e.m., $n=5$ for each value. All values are significantly different in the treated group after treatment.

or inflammatory response. Normal numbers of lymphocytes and histiocytes were observed. Myocytes had normal-sized nuclei, normal sarcoplasm and endomysium (Figure 6a).

The major feature following cryotherapy was severe congestion of all vessels associated with myocyte damage (vacuolation and cross-banding; Figure 6b). In addition, at 4 h after cryotherapy there was evidence of interstitial oedema and white cell accumulation.

### Discussion

The major effect of cryotherapy in normal striated muscle was reduced blood flow followed by a brief period of reperfusion, with complete vascular stasis eventually observed. Macromolecular leakage occurred from all vessels, and mirrored the fluctuations in blood flow. Transient changes in vessel diameters were also observed. The histology confirmed the *in vivo* observations of vessel congestion and muscle damage.

The alterations in blood flow in the cremaster microcirculation following cryotherapy are consistent with previous studies of the hamster cheek pouch microcirculation (Rabb *et al.*, 1974). A biphasic pattern of haemostasis was observed in both the cremaster and the hamster cheek pouch, with complete stasis in all vessels immediately after cryotherapy. This was followed by reflow in some larger vessels 30 min after treatment, which ceased again 60 min following cryotherapy.

**Figure 4** Percentage change in interstitial fluorescence surrounding a venule in controls (O) and treated (●) animals before and after cryotherapy; mean ± s.e.m., $n = 5$ for each value. All values are significantly different in the treated group after treatment.

**Figure 5** Percentage change in interstitial fluorescence surrounding the post-capillary venule in controls (O) and treated (●) animals before and after cryotherapy; mean ± s.e.m., $n = 5$ for each value. *Significant differences.

Rabb *et al.* (1974) found that 20 min after freezing diminished blood flow was associated with the appearance of circulating emboli of aggregated platelets lodging preferentially on the venous side of the microcirculation. Electron microscopy of the tissue at this time demonstrated endothelial cell damage: rupture of the plasma membrane with large vacuoles between the intra- and extravascular components. The vessel lumina were congested with lysed red cells, platelet aggregates and cell debris. At 60 min after treatment, flow ceased in both cheek pouch and cremaster without any visible embolic obstruction. In some animals circulating emboli were observed in the cremaster within the initial 15 min following freezing. These lodged in the venules, but cleared when flow was re-established. The histological analysis of tissue from the present study, 1 and 4 h after treatment, also demonstrated sarcoplasmic vacuolation with vessel congestion but no evidence of thrombi or emboli.

The alterations in cremaster muscle blood flow using *in vivo* microscopy are consistent with laser Doppler flowmetry measurements of red cell flux in the normal rat liver and in liver tumour following cryotherapy (Brown *et al.*, 1993). These studies demonstrated a substantial reduction in blood flow immediately after freezing, followed by a brief period of reperfusion, and then further cessation of flow which was maintained for 8 h in both tumour and normal liver. However, by 24 h red cell flux had returned to pretreatment levels in tumour and normal liver (Brown *et al.*, 1993).

The effect of cryotherapy on blood vessel diameters was transitory and only occurred in the larger vessels. The decrease in vessel diameter may be due to vasoconstriction, particularly as the diameter had returned to pretreatment values 30 min following treatment. However, damage to the endothelial cells and the deposition of mural thrombi may obscure the image of the vessel wall and produce an apparent decrease in vessel diameter. The thrombi may subsequently dislodge during the reperfusion observed (30 min) following cryotherapy, restoring the diameters to pretreatment values.

**Figure 6** **a**, Micrograph of the cremaster muscle 1 h following sham treatment (× 375). **b**, Micrograph of the cremaster muscle 1 h following cryotherapy, illustrating congestion of the vessels (A) and cross-band necrosis of the muscle (B; × 375).

Although there was no histological evidence of thrombi between 1 and 4 h after cryotherapy, thrombi were only observed in the first 60 min following treatment in both the cremaster and the hamster cheek pouch (Rabb et al., 1974).

The mechanisms by which cryotherapy induces alterations in blood flow and vessel diameters in the cremaster muscle are unknown. Systemic factors affecting the microcirculation, heart rate, blood pressure and body temperature remained constant throughout the experimental period. The changes in blood flow and vessel diameters in the 60 min following cryotherapy may be explained by the emboli observed. However, the cessation of blood flow in the later stages of the experiment are difficult to explain, since neither circulating emboli nor vasoconstriction were observed. Thus the effects of freezing may result in damage to the red blood cells, causing deformation and lysis, or direct endothelial cell damage to the vessel walls, increasing macromolecular leakage and haemoconcentration. Classical clotting mechanisms have previously been excluded as a mechanism by which vessel diameters alter and blood flow ceases, since cessation of blood flow is still observed after freezing in the presence of heparin (Quintanilla et al., 1947). Histamine and 5-HT released from mast cells and causing either constriction or dilation have been suggested as possible mediators affecting blood flow, although as yet there is no experimental evidence that freezing triggers the release of such factors.

A variation in the temporal pattern of macromolecular leakage from different vessels was observed following cryotherapy, which may be explained by the fluctuations in blood flow in the same vessels. In the large arterioles the initial increase in interstitial fluorescence was followed by a decrease at 30 min, when there was no blood flow to the vessel and hence the supply of fluorescent albumin may have ceased. An increase in the level of interstitial fluorescence surrounding the arterioles corresponded to the return of blood flow between 30 and 60 min after treatment, followed by a gradual reduction in fluorescence levels as cessation of blood flow occurred. These changes were mirrored in all observed arterioles and venules. However, despite the complete cessation of flow in post-capillary venules following cryotherapy the level of interstitial fluorescence surrounding these vessels continued to increase throughout the experimental duration.

Possible mechanisms for the increase in macromolecular leakage observed immediately following cryotherapy may be due to damage to the endothelium or basement membrane or pericyte damage or contraction. This damage may result in the release of histamine and 5-HT via mast cell degranulation, leading to increased vascular permeability, although there is no evidence for the release of these mediators. Leucocyte margination, migration and activation could result in the release of vasoactive leukotrienes capable of inducing leak. Histological examination of the frozen tissue demonstrated lymphocyte infiltration, possibly as a result of the acute inflammatory response induced by cryotherapy. This may contribute to the macromolecular leakage observed later in the study but does not explain the pronounced initial effect.

In summary, we have demonstrated that cryotherapy appears to induce macromolecular leakage from all vessels, a transient reduction in arteriolar and venular diameters and cessation of flow immediately after treatment, followed by a period of reflow before complete stasis occurs. All of these factors may contribute to cell death and tumour necrosis following cryotherapy. These and previous studies therefore demonstrate that cryotherapy induces a reduction in blood flow and microcirculatory arrest in tumour and normal liver (Brown et al., 1993), normal striated muscle and the hamster cheek pouch (Rabb et al., 1974). The vascular supplies of normal skeletal muscle and normal liver are different, but the gross changes observed within the vasculature following cryotherapy are similar. Although tumours may have an immature microcirculation and be relatively poorly vascularised compared with liver and skeletal muscle, we have demonstrated significant reductions in blood flow in normal liver, implanted liver tumour and normal striated muscle in the rat. It has also been shown that normal and tumour vessel permeability is increased after cryotherapy (Ikekawa et al., 1985), demonstrating that cryotherapy appears to have similar effects in normal tissues and tumours.

The financial assistance of Trent Regional Health Authority, Special Trustees, and the Yorkshire Cancer Research Campaign is gratefully acknowledged. We would also like to thank Mrs J. Globe for preparing the histology and Dr D. McKenna for interpreting the results.

### References

ADSON, M.A. (1987). Resection of liver metastases – when is it worthwhile? *World J. Surg.*, **11**, 511–513.

BAEZ, S., (1973). An open cremaster muscle preparation for the study of blood vessels by *in vivo* microscopy. *Microvasc. Res.*, **5**, 384–394.

BAYJOO, P. (1992). *Cryosurgery and the Immune System.* MD Thesis, University of Leeds (submitted).

BOWERS, W.D., HUBBARD, R.W., DAUM, R.C., ASHBAUGH, P. & NILSON, E. (1973). Ultrastructural studies of muscle cells and vascular endothelium immediately after freeze–thaw injury. *Cryobiology*, **10**, 9–21.

BROWN, N.J., BAYJOO, P. & REED, M.W.R. (1993). Effect of cryosurgery on liver blood flow. *Br. J. Cancer*, **68**, 10–12.

CADY, B. & MCDERMOTT, W.V. (1985). Major hepatic resection for metachronous metastases from colon cancer. *Ann. Surg.*, **201**, 204–208.

CHARNLEY, R.M., DORAN, J. & MORRIS, D.L. (1989). Cryotherapy for liver metastases: a new approach. *Br. J. Surg.*, **76**, 1040–1041.

FARRANT, J. & WALTER, C.A. (1977). The cryobiological basis for cryosurgery. *J. Dermatol. Surg. Oncol.*, **3**, 403–407.

FINDLAY, I.G. & MCARDLE, C.S. (1986). Occult hepatic metastases in colo-rectal carcinoma. *Br. J. Surg.*, **73**, 732–735.

GAGE, A.A., GUEST, K., MONTES, M., CARUANA, J.A. & WHALEN, D.A. (1985). Effect of varying freezing and thawing rates in experimental cryosurgery. *Cryobiology*, **22**, 175–182.

IKEKAWA, I., ISHIHARA, K., TANAKA, S. & IKEDA, S. (1985). Basic studies of cryochemotherapy in a murine tumour system. *Cryobiology*, **22**, 477–482.

KUFLIK, E.G. & GAGE, A.A. (1992). *Cryosurgical Treatment for Skin Cancer.* Igaku-Shoin: New York.

MCINTOSH, G.S., HOBBS, E.F. & O'REILLY, A.P. (1985). *In situ* freezing of the pancreas and portal vein in the pig. *Cryobiology*, **22**, 183–190.

MASTERS, A., STEGER, A.C. & BOWN, S.G. (1991). Role of interstitial therapy in the treatment of liver cancer. *Br. J. Surg.*, **78**, 518–523.

MILLER, F.N., JOSHUA, I.G., ANDERSON, G.L. (1982). Quantitation of vasodilator-induced macromolecular leakage by *in vivo* fluorescent microscopy. *Microvasc. Res.*, **24**, 56–67.

NEEL, H.B., KETCHAM, A.S. & HAMMOND, W.G. (1971). Ischaemia potentiating cryosurgery of primate liver. *Ann. Surg.*, **174**, 309–318.

QUINTANILLA, R., KRUSEN, F.H. & ESSEX, H.E. (1947). Studies on frostbite with special reference to treatment and the effect on minute blood vessels. *Am. J. Physiol.*, **149**, 149–161.

RABB, J.M., RENAUD, M.L., BRANDT, P.A. & WITT, C.W. (1974). Effect of freezing on the microcirculation and capillary endothelium of the hamster cheek pouch. *Cryobiology*, **11**, 508–518.

RAVIKUMAR, T.S., KANE, R., CADY, B., JENKINS, R., CLOUSE, M. & STEELE, G.D. (1991). A 5 year study of cryosurgery in the treatment of liver tumours. *Arch. Surg.*, **126**, 1520–1524.

WHITTAKER, D.K. (1975). Ultrastructural changes in the microvasculature following cryosurgery of oral mucosa. *J. Periodont. Res.*, **10**, 148–157.

WHITTAKER, D.K. (1984). Mechanisms of tissue destruction following cryosurgery. *Ann. RCS Engl.*, **66**, 313–318.

ZHOU, X., TANG, Z., YU, Y. & MA, Z. (1988). Clinical evaluation of cryosurgery in the treatment of primary liver cancer. *Cancer*, **61**, 1889–1892.